# Everyday Resistance in the U.K.’s National Health Service

**DOI:** 10.1007/s11673-023-10274-3

**Published:** 2023-09-15

**Authors:** Ryan Essex, Jess Dillard-Wright, Guy Aitchison, Hil Aked

**Affiliations:** 1 Institute for Lifecourse Development, University of Greenwich, Old Royal Naval College, Park Row, London, SE10 9LS UK; 2 Elaine Marieb College of Nursing, University of Massachusetts Amherst, Amherst, USA; 3 International Relations, Politics and History, Loughborough University, Loughborough, UK; 4 Medact, London, UK

**Keywords:** Resistance, Protest, Health, Healthcare, Activism

## Abstract

Resistance is a concept understudied in the context of health and healthcare. This is in part because visible forms of social protest are sometimes understood as incongruent with professional identity, leading healthcare workers to separate their visible actions from their working life. Resistance takes many forms, however, and focusing exclusively on the visible means more subtle forms of everyday resistance are likely to be missed. The overarching aim of this study was to explore how resistance was enacted within the workplace amongst a sample of twelve healthcare workers, based in the United Kingdom; exploring the forms that such action took and how this intersected with health and healthcare. In depth-interviews were conducted and results were analysed utilizing Lilja’s framework (2022). Our findings suggest that resistance took a number of forms, from more direct confrontational acts, to those which sought to avoid power or which sought to create alternative or prefigurative practices or norms. These findings speak to the complexities, ambiguities, and contradictions of resistance, as carried out by healthcare workers in the workplace. While many acts had clear political motives, with issues like climate change in mind for example, participants also described how the act of providing care itself could be an act of resistance. While saying something about our participants, this also said something about the healthcare systems in which they worked. These findings also raise a range of normative issues. Perhaps needless to say, there appears to be substantial scope to expand and interrogate our findings and apply the idea of resistance to health and healthcare.

## Introduction

When it comes to health and healthcare, resistance is a remarkably understudied phenomenon. Even the most visible forms of protest, for example blockades and sit-ins, have generated little discussion in the health disciplines, leaving a range of conceptual and normative questions unanswered. Beyond these more visible forms of action there is reason to believe that resistance as it relates to health occurs far more frequently, day-to-day and out of sight (Scott 1989). This type of resistance has been labelled “everyday resistance.” Political scientist James Scott first coined this term, contending that focusing only on visible events risked missing more subtle acts of resistance, such as non-cooperation, feigned ignorance, or humour that occur in day-to-day life. Scott also saw everyday resistance as important because it helped to explain the long-term potential for political change, showing how resistance occurs below the surface, even when it appears as if few people are resisting (Scott 1989).

In the context of healthcare, resistance has been defined as “any act, performed by any individual (or collective) … that is a response to power, most often in opposition to contentious, harmful or unjust rules, practices, policies or structures” (Essex 2021, 480). Resistance has a complex relationship with health. We can find examples of health motivating acts of resistance (Redhead 2021), acts of resistance that have implications for health (Zalakeviciute et al. 2021), and even health itself utilized in acts of resistance, such as in cases of self-harm (Aitchison and Essex 2022). Beyond having important implications for health and healthcare, resistance also raises a range of normative questions, many of which remain unexplored. This discussion and the above definition of resistance are far from definitive or final however, and resistance remains a controversial and contested concept. Points of particular contention relate to its relationship to power and whether intent is needed for an act to be considered resistance (Hollander and Einwohner 2004). One further point of contention has been the relationship between everyday and more public acts of resistance. Traditionally, everyday resistance has been conceptualized as individual and hidden, contrasted with more collective and public actions. In contrast, Lilja (2022, 209) argues that “many of the resistance practices that we see today are neither public and mass-organized nor individual and hidden” and that because of this, the relationship between everyday and other forms of resistance “do not cover the complex acts and relations of power and resistance.” Lilja contends that not all individual acts are hidden, providing the example of Greta Thunberg’s School strike for climate; an individual act which received substantial public attention. We can find parallels here with whistleblowing in healthcare, acts which are often carried out by individuals that become “hypervisible” (Perron, Rudge, and Gagnon 2020). We can also see that not all collective acts of resistance are public, for example, Jewish resistance throughout the Holocaust, including the public health systems developed in Jewish ghettos throughout Eastern Europe (Longacre, et al. 2015) and Chicago’s JANE collective, a group who provided illegal abortion care in the 1960s (Kline 2010; O’Donnell 2017).

Proposing an alternative to capture these different forms of resistance, Lilja (2022) and Baaz, et al. (2021) suggest resistance could be categorized in three ways: avoidance resistance, breaking resistance, and constructive resistance. Avoidance resistance seeks to avoid power, this may be through disguise or by masking the act itself, it may be through maintaining anonymity; such acts can be both individual and collective. Breaking resistance refers to action that confronts power, acts such as blockages, strikes, or marches. Not all resistance however is “oppositional.” Constructive resistance refers to a form of resistance that “constructs ‘alternative’ or ‘prefigurative’ social institutions or discourses” (Lilja 2022,210). Such resistance includes building new institutions or norms, for example. These forms of resistance are not mutually exclusive, since any one action can combine different elements of each. An occupation of public space, such as a building or square, organized on principles of horizontal democracy, for example, combines elements of both breaking and constructive resistance.

When it comes to action from healthcare workers, we can find examples of each type of resistance. Most commonly reported are breaking resistance: collective, public acts, such as strikes (Essex et al. 2021), marches (Amin 2022), and even civil disobedience (Taylor 2021)—perceptible forms of resistance, legible as resistance to those outside the health professions. We also don’t have to look far to find instances of individual public protest, such as Dawn Wooten, a nurse who publicly blew the whistle on sterilization practices in an Immigration Customs Enforcement facility in the United States (Remnick 2022). While less common, we can find clandestine individual (Essex 2022) and group actions (Longacre et al. 2015) that fit avoidance resistance; however when looking at how such resistance is enacted in the workplace, examples are difficult to locate. Studies have detailed how health workers have resisted workplace bullying (Leaver 2019) and the bureaucratization and managerialization of healthcare (Rudge 2011). For example, amongst eleven clinicians Saraga, Boudreau, and Fuks (2019) found that participants often broke the rules when it came to generally accepted norms of practice, such as clinical guidelines, when these norms were perceived as barriers to accomplishing clinically necessary interventions. Similarly, results from an ethnographic study of a hospital implementing patient safety systems described physicians pushing back against management efforts to restrict clinical freedom and professional autonomy, resisting bureaucratization through refusal to engage in reporting and the appropriation of managerial spaces (Waring and Currie 2009). Beyond these examples, resistance in the workplace takes more diverse forms and is motivated by a range of issues. In Australia, Mainey, O’Mullan, and Reid-Searl (2022) found that nurses who provided abortion care undermined laws, systems and individuals who sought to impose barriers on access to abortion. Such acts also occur in far less politically charged settings, for example a body of work by Shutzberg (2019) explored strategies employed by Swedish GPs to increase the likelihood that patients’ sickness certificate would be approved by the Swedish Social Insurance Agency. These strategies included exaggerating symptoms, omission, and the utilization of “buzzwords.” This small body of work speaks to the complex nature of resistance and raises several questions not only about these acts but the systems and structures that shape the delivery of care, the simultaneous power and powerlessness that healthcare workers hold in their roles and what the potential benefits and trade-offs are in understanding such action as resistance.

This study seeks to build on the above literature, which has largely focused on “events” and narrowly specific contexts (for example abortion care in Australia). Rather than specific contexts, here we explore acts of resistance amongst a sample of U.K. healthcare workers more generally. While all reported engaging in various forms of non-violent action, here we focus on resistance within or in relation to the workplace as a form of everyday resistance. The overarching aim of this study was therefore to explore the ways that resistance is enacted *within* the workplace, exploring the forms that such action took and how this intersected with health and healthcare. We hope that documenting such actions will contribute to a more nuanced understanding of resistance as undertaken by healthcare workers. We also see this work as building important context for future discussions about the significance of resistance in healthcare settings, in particular if and when resistance is morally justified—or even obligatory.

## Methods

### Procedure and Participants

This study recruited healthcare workers based in the United Kingdom. Participants’ contact details were sought through a previous study which sought to explore their involvement in and understanding of non-violent resistance (Essex, et. al. 2022). Participants were originally recruited through the Medact (https://www.medact.org/) member database. Medact is a non-profit campaign organization primarily made up of healthcare workers, with a primary focus on peace and security, climate and health, economic justice, and health and human rights. For this reason, our sample is unlikely to be representative of the broader healthcare community in the United Kingdom. In saying this, however, given the nature of this sample, they were in an advantageous position to provide insight in relation to our research questions, namely in exploring everyday resistance and the forms it took in the day-to-day delivery of care. Forty-one participants were contacted via email with twelve participating in in-depth semi-structured interviews. Participants were physicians, nurses and other healthcare workers. Five participants were retired, most within the last five years, the sample contained a mix of junior, mid-career, and senior clinicians; all had engaged in acts of resistance outside the workplace. The majority of participants worked or had worked within the NHS with a smaller number working for private companies that provided services for the NHS. The interview schedule was informed by the above study and asked questions related to resistance within the workplace, along with several more wide-ranging questions related to the justification of resistance and actions outside the workplace. Interviews were conducted online, throughout late 2021.

### Analysis

Interviews were recorded and transcribed. Transcripts were reviewed and data categorized according to Lilja’s (2022) framework outlined above. Acts of resistance were identified and categorized as avoidance, breaking, or constructive resistance. The analysis therefore focused on the actions undertaken by these healthcare workers. While we were not focused on participants’ reasons for undertaking these actions, their intent, or the barriers and facilitators for such action, we inevitably touch upon some of these issues below in explaining the acts of resistance that were disclosed. RE carried out initial coding, with all authors having input into the final coding presented below. Disagreements were discussed until consensus was reached.

### Ethical Approval

Ethical approval for this study was granted by the University of Greenwich Research Ethics Committee (UREC/21.1.6.10).

## Results

A range of actions were reported by participants. These varied from more open and public acts (i.e. acts that occurred in the workplace in view of the public or in public spaces) that confronted power to less visible and more subtle day-to-day acts. These acts sometimes confronted power directly, while at other times they sought to avoid direct opposition. While less common, participants also reported engaging in constructive acts of resistance. Each will be explained below.

### Avoidance

Participants detailed a number of actions that could be considered avoidance resistance, that is, acts that did not directly challenge power or did so through disguise. The first form of avoidant action involved undermining or subverting rules, practices, or procedures. A number of participants spoke of actions related to the enforcement of migration controls within the NHS. Many participants simply refused to ask about the migration status of their patients, while others undermined staff who sought to check migration status. One participant provided this account from when they were a student:

*I remember someone was trying to fill out a form for someone when I was a student, for someone about their immigration status in healthcare, and so I hid the form so they couldn*’*t do it. (P16)*

Two further examples referred to the NHS’s approach to identifying and charging those who have undocumented migration status. One participant spoke about the importance of context in navigating acts of resistance. They noted how, when they worked at a GP surgery, providing care for undocumented patients was not considered subversive or controversial. Another described resistance from a colleague who actively subverted her job role, while working as a manager for “overseas visitor charging” understanding this as an opportunity to “persuad[e] the Trust not to charge people if they couldn’t afford it” (P17) 1. This stood in stark contrast, in the participant’s recounting, to others in the same role whose sole occupation was extracting payment from the euphemistically termed “overseas visitors.”

Other participants described experiences during the COVID-19 pandemic. One healthcare worker provided an account of his experience working in contact tracing. The system that was being used did not allow potential contacts to return calls and when dialling out, the prefix of the phone number often meant people avoided answering—as a result, the system was largely ineffective. Counter to the participants’ contract, he used his personal mobile phone for calls, allowing people to return his calls. He went on to describe it as “not a major act of resistance, but it was actually breaching the contract … but the other system didn’t work … I was getting through to people using my own system” (P11).

Participants also spoke about a range of other actions. At least two participants mentioned leaving their roles. One participant identified changing career paths as an act of resistance, becoming a health visitor which provided far more autonomy than a traditional nursing role, noting that becoming a health visitor was “in some ways a rebellion against the standard … You’re on your own job more or less” (P18). One participant, while apprehensive about the potential repercussions about airing political views would still allude to these using humour to offset the expressed perception that “I wouldn’t feel like I can have an open and honest conversation with a patient about my political beliefs” (P9). Communicating in this way, using humour as a buffer could be understood as avoidance, in that humour was used to sidestep any potential conflict or even have this recognized as an open challenge to the government. Notably, this participant also engaged in more open, breaking acts of resistance with other staff, which will be discussed below. Finally, a further participant also reported what was seemingly benign activity motivated by their concern for climate change. This involved turning off colleagues’ computers at the end of the day. While initially this started as nonconfrontational, after being identified and asked to stop, the participant found themselves with the dilemma as to whether they persevered with this action, at the risk of becoming increasingly confrontational.

*But there were activities we would do about sustainability around the hospital. Whether it*’*s turning the lights off or turning off people*’*s computers or whatever. I*’*ve got trouble for that before, at the end of the day going around and turning off* … *ten computers, which people just leave on overnight. I*’*ve been warned about that and I find that extraordinary. We*’*re in a public environment and surely we all have a duty to perhaps save energy, save hospital electricity bills, but also save carbon and try and start behavioural patterns to make it the norm for people to shut things down when they leave their office. But I*’*ve had comeback from that and complaints from management … It*’*s bizarre … it*’*s difficult and it*’*s certainly caused friction in the workplace*.* (P41)*

One further example provides contrast here, detailing avoidant action that broke the law. Like the above examples, it again appears to (at least partially) be motivated by concern for patients, or as having “empathy with dying people” (P30) as described by the participant. This participant spoke of working in a hospice, shortly after smoking was banned indoors.

*When smoking was banned in hospitals, in hospices, hospices were exempt from that law. But we had a lot of smokers. But in our hospice, we had smoking areas, and you had to go out into the garden if you wanted to smoke. But there were people who were bed bound who were desperate for a fag and we were not allowed and it was a hospice that had been newly refurbished and that every room had a balcony or you could go out onto the patio … they were not allowed to smoke out there because the smoke wafted around and people in other rooms were irritated even outside particularly in the summer … everybody knew those rules were broken and I think it’s slightly different in the hospice because it’s such a small community compared to a hospital … We even used to put gloves over the smoke alarms so that the patient could have a have a fag in the room and the alarm wouldn’t go off. Because that’s just empathy with dying people who are desperate for a cigarette. (P30)*

Assisting patients to violate a ban on smoking may at first sight seem like a morally ambivalent instance of resistance. But the case points to how, within a rule-bound healthcare setting, there are other concerns at play such as respect for patient autonomy and empathy for a dying person’s wishes; it could also be argued that in this case, that any harm to the patient was negligible and the only potential harm was to staff.

### Breaking

A number of actions were also identified that could be considered breaking resistance, that is, resistance that directly challenged power through more open forms of protest. This form of resistance was far less common, and mainly came in the form of verbal challenges. For example, a number of participants reported challenging other staff, mainly if they had been disparaging to patients. While this participant was reluctant to speak about their political beliefs with patients, they spoke about openly challenging other staff, including those who were in more senior roles.

*… where staff members have been disparaging or derogatory about patients because of their circumstances or, suggested that because they’re, for example, a substance user that they have xyz traits… So I think challenging colleagues and seniors on those things is something I have done and I will continue to do*.* (P9)*

Another participant recounted an example from early in their career, where they had challenged a more senior member of staff who appeared to wish to punish a patient following a suicide attempt. The punishment took the form of clinically unnecessary and painful blood glucose fingersticks. Despite being earlier in their career, the participant reported challenging the senior physician “[…] in front of the patient” raising concerns that the fingersticks were not clinically indicated. As a result the “consultant kind of caved and agreed to do something a little bit more clinically indicated” (P33). This resulted in poor treatment of the participant from the consultant for some time after the confrontation. In addition to verbally confronting colleagues, other healthcare workers challenged patients “who are racist towards black staff” (P30). Here, resistance takes a more head-on approach in addressing a problem or issue, however in terms of care, it also could be seen as more ambiguous. That is, participants sometimes directed their opposition at decision-makers with authority over them in the name of upholding standards of care that the institution itself purports to be bound by. Whether or not this counts as resistance is ambiguous, because although power is being challenged, this is being done to uphold publicly acknowledged professional standards.

Beyond verbal challenges, breaking resistance took a number of other forms. One healthcare worker, who was actively opposed to the privatization of the NHS, used the NHS pharmacy leaflet rack as a site to resist pharmacy privatization. Every time the participant passed the rack, they “put leaflets in” such as a flyer bearing the slogan “Boots loots” which “explained the background to [pharmacy company] Boots being involved” in healthcare privatization (P17). This went on for months, eventually leading to the removal of the rack. The same participant employed another tactic that sought to challenge power by submitting freedom of information requests, forcing more accountability in relation to Trust finances. Seeking information on just how much was invested, the participant “asked senior management how much they’d spent on those management consultants.” Upon their refusal to answer directly, the participant filed a “Freedom of Information request and they said that they couldn’t release the information because it was commercially sensitive” (P17). Unfaltering, the participant persisted, going to the Information Commissioners Office. “Then after nine months, they forced the Trust to release the figure and it was about 800,000, it was some ridiculous figure” (P17). Rather than falling back or avoiding confrontation, this participant engaged in a form of breaking resistance that openly challenged managerial and bureaucratic opacity.

### Constructive

While less common, a number of participants discussed what could be best described as constructive forms of resistance—that is to say, the promotion of alternative social relations based on desired ideals. These most frequently took the form of providing care, that is, providing care that they felt was patient-centred, against systems and structures that oppressed and dehumanized patients. This included steps to move away from medical terminology recounted by one participant who noted, “I think letters when you’re writing to patients can often seem quite pathologizing. I’ve tried to just write, most of my letters to the patient, so I’m speaking to them directly, and not about them” (P37), creating the opportunity for connection with care-seekers while cultivating an affirming understanding. A number of participants identified that providing care itself was an act of resistance. Sometimes this manifested as simply honouring the dignity and worth of people seeking care. One participant noted that “there’s quite a lot about trying to make sure we treat people decently and with respect, that actually does alter the outcome” (P11). Other times, resistance took the form of carving out time to sit with patients to ensure their needs were met, irrespective of time demands or extrinsic expectations of productivity. One participant related experiences in a walk-in clinic where “a lot of vulnerable people come through the door and […] you’re meant to take twenty minutes, half an hour, with each patient at most, but I would take two hours if the patient needed two hours, and I would take the time to find the interpreter if it took time” (P9).

Another clinical example of constructive resistance was reported by a participant who understood the necessity of sanctuary within the clinical environment, noting that, “we were doing our own thing that, you know, it wasn’t a revolutionary thing we were doing like talking therapies and such like, with nice, comfortable furniture, and people came off the wards. It was like a day hospital within the hospital” (P25). This constructed a small and fragile “bit of a sanctuary within the hospital, and people were treated right … it made some contribution to positive outcomes” (P25). Sometimes, the constructive intervention is simply offering another schema or framework for understanding the condition of a care-seeker. This participant also shared that they frequently work to reframe mental health challenges with families, helping them understand that “there’s different ways of thinking about mental health and mental distress, and that they can have a role in supporting people differently that isn’t just nagging them to take the medication” (P25).

Beyond these accounts of action that involved patients, participants discussed a number of examples where they had engaged in constructive or prefigurative acts. These included efforts to reduce their carbon footprint and to democratize the workplace. One participant described their efforts to catch public transport while at work: “[e]very time someone asked me if I’m driving, I say no … I’m going to get the bus—I’m trying to save the planet. I do it kind of jokingly. But I’ve noticed some of my colleagues are now walking and getting public transport more because they see what I’m doing” (P33).

A final example of constructive resistance comes from health education. Recognizing the stifling limitations imposed by regulatory strongholds on health professions education, one participant reported that, “ … over the years, we tried to develop a sort of alternative curriculum. So, one of the things that happened in nursing is the regulatory bodies and everything that made it a very competency organized curriculum, which squeezed out things like sociology.” This left a clear mission: the participant and colleagues sought to “put that stuff back in front of the students” by asking them “would you like to come to another series of seminars that’s on this radical stuff like anti-psychiatry and the critiques of anti-psychiatry, and like critical accounts of medication regimes … ” (P25). Education is an important site of development for identity in the health professions and adding back perspectives jettisoned by regulatory capture constitutes a form of constructive resistance.

## Discussion

This study sought to explore the ways healthcare workers resisted within the workplace, documenting how healthcare workers themselves understood what resistant meant, the forms this action took and how it intersected with health and healthcare. To begin to organize our data, we turned to Lilja’s (2022) formulation of resistance, categorizing acts as avoidance, breaking, and constructive forms of resistance. Perhaps the first and least surprising result was that acts of resistance were identified as frequent occurrences by health workers and took a range of forms. These acts ranged from arguably mundane and avoidant acts to more confrontational, public, and even illegal acts. These findings show a number of similar forms of action, identified by Shaw, and colleagues (2018) like verbal challenges and modelling behaviours and Mainey et al. (2022) like subversion as a means to place patient interests first. While no participants discussed “doctoring” documents, parallels can also be found with Shutzberg (2019) and other work that has explored how health workers have responded to bureaucratic and managerial encroachments into clinical care; in this study participants utilized a variety of tactics to navigate and undermine systems and structures that would otherwise harm health or undermine their clinical judgement; beyond this however, participants also reported a wide range of actions and motivations.

This research has several implications for ongoing discussion in the resistance studies literature. These findings reinforce Shutzberg’s (2021) observation that resistance, when carried out by healthcare workers in this study was not only readily explained by their relative powerlessness but by the fact that they had power; that healthcare workers are both the subject and object of exerted power. Our participants were a diverse group of healthcare workers, physicians, nurses, and others at varying stages of their career or retired. These factors, along with a range of other contextual factors influenced the forms of resistance that were enacted. That is, we can see how a number of participants were often disempowered by systems, structures, policies, or other individuals within the workplace, while at the same time utilizing their position to resist, whether this be openly challenging or undermining policy or other staff for example, or using their position to educate others (see below for the discussion on the implications of these findings in understanding what this means for healthcare systems). While we have not reported any demographic details of our participants because of the sensitive nature of these interviews and to maintain participant anonymity, we did note some differences in between those starting out in their career as compared with those who were more established. For example, while a number of those earlier in their career engaged in more confrontational and disruptive actions outside the workplace, they would adopt more avoidant or constructive forms of resistance in the workplace. At the same time, it appeared those who were more senior or further into their career were more willing to leverage their relative power and engage in more overt acts of resistance in the workplace. This however was not fixed, as we detailed above, participants also shifted the form that their resistance took, with a number engaging in acts of breaking, avoidance, and even constructive acts of resistance. In saying this, care should be taken in generalizing these observations, our sample cannot be considered representative of the broader healthcare community in the United Kingdom or elsewhere. This is, however, an issue that deserves greater exploration, namely how health workers leverage their relative power in the workplace. Our conclusions here are also somewhat speculative. It is plausible that those more senior may have been more or less likely to speak out because of the power they held. They may have been more likely to engage in confrontational action because the consequences of such action may be less serious but at the same time less likely to do so because they were in positions to influence systemic change, rather than having to engage in such action.

Following this point, our results also speak to the dynamic and relational nature of resistance. The above findings speak to how resistance shifted, from action which initially attempted to avoid power, to more confrontational action. This was discussed by the participant who turned off colleagues’ computers at the end of the day, a seemingly benign act which created tension at work after they were caught; the participant continued to turn off computers. This raises interesting questions about when avoidant acts become more confrontation, perhaps showing how visibility may turn an act of avoidant resistance into one that more directly challenges power. Similar things could be said of constructive acts: at what point does a two hour consultation with a vulnerable patient become a more direct challenge to management or other colleagues who would prefer shorter appointments? Interestingly, participants also disclosed acts that would be considered resistance elsewhere, being actively encouraged and supported. As one participant revealed, the GP practice in which they worked did not ask for identification or question entitlement to healthcare, elsewhere in other environments we saw participants employing a number of tactics to challenge and undermine efforts to identify patient eligibility for care.

While not the focus of this study, a number of participants touched upon motivation and intent. In terms of intent, a number of participants felt that when it came to patient care they were not engaging in acts of resistance, or that their actions were not political, but simply working within existing systems, utilizing the grey area that they were afforded to provide care in the face of barriers. This will be discussed below, but it does raise questions such as whether intent is needed for an action to qualify as resistance or whether the outcome of the action is more significant, for example. Our impression from these interviews was that participants engaged in acts of intentional and unintentional resistance and that to understand this it was not only important to understand participants’ intent or motivation but also, how these actions were “understood, constructed and located by others” (Lilja 2022). It is also likely that many will see what we have labelled acts of resistance as simply providing care or fulfilling professional duties. We will discuss below why we feel that these acts could be considered resistance.

This research also has several implications in understanding the role of healthcare workers and the systems in which they work. A question that is naturally raised is could an act be considered both resistance and generally accepted care? Could an act be considered both resistance and any number of other concepts that are found in the health and bioethics literatures? In regard to this first question, it seems possible that the act of providing care could also be considered an act of resistance. Whether this is the case however will depend on a range of factors. For example, if structures, systems, or norms enable and encourage practice that is ethical and patient-centred, providing care may not be an act of resistance. However, if this is not the case and these same structures, systems, or norms undermine care, simply making the time and space to see patients could be an act of resistance. On the second question, we also believe that acts of resistance could be conceptualized other ways. For example, one may instead label some of the examples as “workarounds.” Workarounds have been described as acts that “circumvent or temporarily ‘fix’ perceived workflow hindrances to meet a goal or to achieve it more readily” and include behaviours such as “violations, deviations, problem solving, improvisations, procedural failures and shortcuts” (Debono et al. 2013, 1). Similarity, it is possible that many of the acts described above could be readily described as “coping” (Shutzberg, 2020); coping with systems, structures or individuals that impact patient care for example. However, even if an act could also be described using different terminology, it may still also be an act of resistance, that is, it could be both resistance and a workaround, it could also be resistance and coping. This then raises a further question, namely the potential impact of using resistance to label such acts. There appear to be a range of potential benefits and trade-offs here in doing so. For example, we may risk overextending the term resistance to an act which should be considered and normalized as care, however potential benefits may include drawing attention to the political nature of care and the fact that care, along with what might seem like mere organizational issues are tied to bigger political/ideological contestations. This also deserves far greater attention than what can be provided here.

A question that follows, relates to what these findings say about the NHS, particularly in light of several participants conceptualizing acts of care as acts of resistance. While these findings provide insight into the reality of delivering care, showing how healthcare workers exercise power and challenge the prevailing notion that care is simply directed by evidence or institutional policies (Saraga et al. 2019), it shows that things are far more complex. It is notable that over the last several decades health worker power has been curtailed and modulated by a range of stakeholders such as hospital management, insurance companies, and the government through law or policy (Numerato, Salvatore, and Fattore, 2012). Beyond this, we find a critical literature that discusses how healthcare systems function to elicit discipline and order (Rudge 2011), as well as a literature critical of the NHS; an organization with a “persistent dysfunctional organizational culture” (Pope 2019). This literature details the detrimental impact of hierarchy on the delivery of care (Brennan and Davidson 2019; Walton 2006) and even more generally, the oppressive nature of healthcare systems and modern medicine. These structural factors arguably explain why a number of participants saw the de-medicalization of language or the act of providing patient care as acts of resistance. That is, resistance in response to a focus on efficiency rather than patient care, tacit and explicit endorsement of behaviours that harm patients and against practices which dehumanize. These finding also say something about how we might begin to look to address these shortcomings, notably that dissatisfaction, amongst other concerns, may manifest in other ways with participants engaging in a range of actions to undermine workplace policy, procedures, or simply in the interests of putting the needs of their patient first. It follows that looking only at open acts of defiance alone does not completely gauge dissatisfaction in the health system or explain deficits in care for example. In saying this however, our participants revealed forms of resistance that could not just be seen as a response to encroachments on their clinical decision-making or for patient well-being; participants engaged in a range of actions that related to broader social and political issues, such as climate change.

Lastly, this research raises several normative and regulatory questions. A longstanding criticism of everyday acts of resistance and particularly avoidant acts of resistance is that they fail to challenge the systems and structures which create the initial conflict. As such, this kind of resistance allows the systems, structures, or individuals to carry on unchecked and in subtle, indirect ways may even facilitate these things. Shutzberg (2021) describes the position on GPs in issuing sickness certificates as one that involved a mix of compliance and resistance. We again found parallels in many of the acts outlined by our participants, where resistance at least partially relied on a façade of compliance. A number recognized that “it wasn’t a revolutionary thing we were doing” (P25) and as noted above, many even questioned if they were in fact engaging in resistance. This is visible in a number of examples, but one particularly salient example comes from the healthcare worker who utilized their own phone when contact tracing during the COVID-19 pandemic. It could be argued that this action not only failed to challenge the inadequate process that was set up but also lent a system, that was widely seen as completely inadequate, a veneer of legitimacy in meeting the need to track and trace those who may have been in contact with COVID-19. More generally, questions are raised about whether this type of action, in very small ways, contributes to the stability and legitimacy of the NHS, a system which has been underfunded for decades, not allowing the true impact of this neglect to be felt. A further interesting example comes from the participant who allowed patients to smoke in their hospice room. This example raises questions about, amongst other things, patient dignity, the law, and professional values. In regard to regulatory issues, while questions have been raised about public protest and the potential impact that this could have on registration, little has been said about acts of resistance within the workplace.

Several limitations should be acknowledged. The focus of this study was relatively broad compared to other studies in this area that have looked at specific issues (i.e. abortion care, professionalism, or issuing sickness certificates). While this has broadly documented acts of resistance, exploring the various forms they take in healthcare settings and outside the workplace, this comes at the cost of some depth. More could and should be made of this data in future studies. These results are not generalizable, nor did we expect them to be. Our sample represents a small portion of U.K. health workers, all of whom were recruited through a campaign organization, focused on issues related to social justice, the environment, and human rights. Thus our sample was unlikely to hold similar views to the broader healthcare community about a range of social and political issues. Finally, we have not discussed other elements of this action in this paper, simply for practical reasons, that any reasonable analysis of the forms of resistance, its motivators, facilitators, and constraints would not have been possible in this paper.

This study speaks to the complexities, ambiguities, and contradictions of resistance, as carried out by healthcare workers in the workplace. The above results raise questions related to the nature of resistance and how it has been conceptualized in the literature but also about the nature of health and healthcare and their intersections with resistance. While many acts were motivated by more clear political motives, such as resisting privatization or concerns about climate change, we found examples where acts of care could also be considered acts of resistance. These acts, while they said something about our participants and how they leveraged their power, also say something about the forces that impact care. Beyond these implications these findings have for discussions on resistance and the NHS, they raise several normative issues, which deserve far greater attention. Needless to say, there appears to be substantial scope to expand and interrogate our findings and apply the idea of resistance to health and healthcare. Several particularly salient issues standout in this respect. Namely, the relationship of resistance and other concepts, workarounds for example and what we gain (or lose) in conceptualizing such acts as resistance. How the relative power and powerlessness of health workers impacts, inhibits, or shapes resistance. What such acts say about the systems in which people work, particularly when health workers conceptualize the delivery of healthcare as an act of resistance. Looking to such acts can also be instructive, not only in identifying flaws or shortcomings in the systems that should support care, but resistance can show us how things could be otherwise, imagining and in some cases demanding better for healthcare workers and patients alike.

## Data Availability

Because of the sensitive nature of this data it will not be made available.

## Footnotes

1 Trusts are organizations within the U.K.’s National Health Service, usually divided along functional or geographical lines.
